# Salvianolic Acid B Inhibited LH2 Expression to Reduce Collagen Synthesis in Pulmonary Fibrosis

**DOI:** 10.1111/jcmm.71168

**Published:** 2026-05-25

**Authors:** Songjun Shao, Shanshan Rao, Silu Hu, Yabin Zhang, Peng Zheng, Chunjie Li, Lichun Zhong, Yunzhi Long, Xin Tian, Xiaoju Tang, Fengming Luo

**Affiliations:** ^1^ Department of Respiratory and Critical Care Medicine, State Key Laboratory of Respiratory Health and Multimorbidity West China Hospital, Sichuan University Chengdu China; ^2^ Department of Respiratory and Critical Care Medicine Guizhou Provincial People's Hospital Guiyang Guizhou China

**Keywords:** collagen cross‐linking, extracellular matrix, lysyl hydroxylase 2, pulmonary fibrosis, salvianolic acid B

## Abstract

Collagen deposition and scar formation are hallmark features of fibrotic diseases. Lysyl hydroxylase 2 (PLOD2/LH2), a key collagen‐modifying enzyme, catalyses lysine hydroxylation at telopeptide sites to promote pyridinoline cross‐link formation, thereby enhancing collagen stability and matrix stiffness. However, the mechanisms regulating LH2 in pulmonary fibrosis (PF) are not fully understood. Here, we identify Salvianolic acid B (SAB) as a potent antifibrotic compound that targets LH2‐associated collagen cross‐linking. LH2 expression was markedly increased in alveolar epithelial cells and fibroblasts during PF, and its silencing attenuated TGF‐β1‐induced fibrotic protein expression. SAB reduced LH2 protein levels and significantly alleviated fibrotic remodelling, as evidenced by restored lung architecture and reduced collagen deposition. Mechanistically, the antifibrotic effects of SAB and LH2 were associated with inhibition of epithelial–mesenchymal transition (EMT), fibroblast‐to‐myofibroblast transition (FMT), and the Wnt/β‐catenin signalling pathway. This study indicates that pharmacological inhibition of LH2 by SAB effectively disrupts collagen cross‐linking and fibrotic progression, offering a promising therapeutic avenue for pulmonary fibrosis.

## 1 Introduction

Fibroblasts are the principal sculptors of extracellular matrix (ECM) architecture. Injury triggers the activation of discrete subpopulations that orchestrate repair, yet persistent activation drives maladaptive fibrosis [1, 2]. Idiopathic pulmonary fibrosis (IPF) is characterized primarily by chronic interstitial lung damage and excessive collagen deposition in the ECM [3]. Currently, Nintedanib and Pirfenidone are the approved drugs for IPF. However, both agents are associated with gastrointestinal, dermatological and cardiovascular adverse effects, and neither has demonstrated a definitive survival benefit [4]. Recent studies have indicated that stem cells derived from the data small airways may alleviate subjective symptoms and enhance lung function in pulmonary fibrosis; but the long‐term efficacy of such interventions necessitates further exploration [5, 6]. The clinical urgency of pulmonary fibrosis mandates the identification of novel molecular targets and next‐generation therapeutics to transform prevention and treatment.

An imbalance in collagen synthesis and degradation is a significant factor contributing to the development of pulmonary fibrosis [7]. Post‐translational modifications (PTMs) in which collagen cross‐links have a substantial effect on the structure and functionality of the ECM [1, 8]. Growing evidence suggests that the ECM instructs fibrosis progression [8]. Collagen PTMs are crucial for ensuring the stability of mature collagen synthesis [9]. Targeting collagen biosynthesis is a promising antifibrotic therapeutic avenue. Masahiko Terajima [10] highlighted the role of LH2 in facilitating collagen modifications that influence collagen stability, organization, and mineralization within MC3T3 cells. In fibrosis, the expression of LH2, the enzyme responsible for the production of telopeptidyl hydroxylysine, is significantly upregulated [11]. It is hypothesized that inhibiting this enzyme may reverse fibrosis without impairing natural repair mechanisms. Research has revealed an increase in PLOD2 gene expression (also known as LH2) in the actively fibrotic regions of IPF tissue, as reported in the Gene Expression Omnibus (GSE169500) [12]. The increased stiffness of IPF tissue is attributed to pyridinoline cross‐linking PTMs. The inhibition of pyridinoline cross‐linking normalized mechano‐homeostasis and limited the self‐sustaining effects of ECM collagen on the progression of fibrosis. Nonetheless, the precise mechanisms by which LH2‐mediated collagen cross‐linking PTMs induce pulmonary fibrosis remain incompletely understood.

Natural products are uniquely suited to treat fibrotic disorders because of their high level of safety and precise efficacy [13]. By integrating the literature and our previous research endeavours, we discovered that SAB could nonselectively inhibit the expression of LH2 [14]. SAB is a highly bioactive component with hydrosoluble properties, and that is the sole monomeric bioactive compound derived from *Salvia miltiorrhiza* (SM). This compound contains seven phenolic hydroxyl groups and has a molecular weight of 718 Da, conferring potent antioxidant, anti‐inflammatory, and antifibrotic effects, and indicating its therapeutic potential in pulmonary diseases [13, 15]. Li reported that network pharmacology analysis predicted potential targets for SAB in the treatment of IPF [15]. Pathway enrichment analysis of codifferentially expressed genes (co‐DEGs) conducted via Metascape revealed associations with the ECM. SAB has been shown to inhibit fibrosis in visceral organs following both injection and intragastric administration [16]. Furthermore, Yadong Fu reported that SAB attenuated liver fibrosis by targeting ECM and inhibiting hepatocyte ferroptosis [17]. Additionally, a study by Qian‐Qian Chen reported that SAB improved renal fibrosis [18]. Despite its antifibrotic properties, the precise molecular mechanisms and targets of SAB have yet to be elucidated. Further analysis of the GEO dataset (GSE169500) revealed that the Wnt/β‐catenin signalling pathway observably associated with IPF. Pirfenidone has been shown to mitigate IPF effects by modulating both the Wnt/β‐catenin and the TGF‐β/Smad signalling pathways [19]. Consequently, the inhibition of Wnt/β‐catenin signalling has emerged as a potentially effective therapeutic strategy for IPF.

In this study, we aimed to investigate the SAB mediating LH2 antifibrotic effects and to further explore whether these effects involve modulation of the Wnt/β‐catenin signalling pathway.

## 2 Materials and Methods

### 2.1 Chemicals and Reagents

Salvianolic acid B (purity exceeding 98%, Catalogue No. N1806) is synthesized by APEXBIO (American). Bleomycin sulfate (CAS No. HY‐17565) was acquired from MedChemExpress (American). Recombinant human transforming growth factor‐beta 1 (TGF‐β1) was purchased from PeproTech (catalogue no. 100‐21; Rocky Hill, NJ, USA). LipofectamineTM RNAiMAX was acquired from Thermo Fisher Scientific Invitrogen (CA92008, USA). Primary antibodies targeting LH1 (ab262947), LH2 (ab184337), LH3 (ab89263), LOX (ab174316), pro‐SPC (ab211326) fibronectin (FN, ab45688), α‐smooth muscle actin (α‐SMA, ab5694) and Collagen Ι (Col Ι A1, ab138492, ab270993) were obtained from Abcam Limited Inc. (Cambridge, UK). Primary antibodies targeting β‐catenin (8480T) and E‐cadherin (E‐ca, 3195T) were obtained from Cell Signaling Technology Inc. (Massachusetts, USA). An antibody against Wnt3a (PSH19‐96) and GSK‐3β (SY02‐71) were obtained from HUABIO Inc. (China). Anti‐GAPDH horseradish peroxidase (HRP)‐conjugated secondary antibody was used. NcmECL Ultra was purchased from Xinmei Biotechnology Co. Ltd. (Zhejiang China). Hydroxyproline and hydroxylysylpyridinoline were procured from Jiancheng Bioengineering, Nanjing, China.

### 2.2 Animal Experiments

Specific‐pathogen‐free male wild‐type (WT) C57BL/6 mice (certificate no. SCXK2020‐034, 6–8 weeks old, weighing 20 ± 2 g) were purchased from Chengdu GemPharmatech Co. Ltd. (Chengdu, China). All the animal experiments adhered strictly to the Provisions and General Recommendations of the Chinese Experimental Animals Administration Legislation and received approval from the Research Animal Care Committee of Guizhou Provincial People's Hospital [Protocol ID 2020‐420]. The animals were housed in a temperature‐controlled (22°C ± 2°C) and humidity‐controlled (50% ± 10%) room. The photoperiod was 12 h light and 12 h dark. The mice were provided ad libitum access to a standard diet and water. The modelling methodology was based on a previous study [20]. The mice were randomly divided into three groups: the control (NS) group, the bleomycin (BLM) group, and the SAB + BLM group. Bleomycin (50 μL, 3.0 mg/kg, diluted in saline) was administered intratracheally (IT) to the mice in the BLM group and SAB + BLM group. In contrast, the mice in the NS group were given equivalent volumes of saline. The first day following intratracheal instillation of bleomycin, the mice were given extra SAB (10 mg/kg body weight) daily through intraperitoneal (IP) injections until 27 days in the SAB + BLM group. The dose and duration of SAB treatment were selected on the basis of previous reports [21]. All the mice were euthanized 4 weeks post‐bleomycin treatment. Lung tissues were either rapidly frozen and subsequently preserved in liquid nitrogen for further examination or perfused and fixed in 4% formalin for a minimum of 24 h at room temperature for immunohistochemical analysis.

### 2.3 Histological Analysis

Idiopathic pulmonary fibrosis (IPF) patient's lung tissues were collected with approval from the Research Ethic Committee of West China Hospital of Sichuan University [2021‐1271]. Lung tissues were fixed with 10% formalin. The samples were dehydrated, cleared, embedded in paraffin, sectioned, dewaxed, hydrated, and subjected to other steps. The sections were subjected to haematoxylin and eosin (H&E) or Masson's trichrome (Masson) staining to assess the intensity of pulmonary fibrosis. Seasoned pathologists assessed the stained sections in a single‐blind manner. Fibrosis outcomes were evaluated via the Ashcroft score [22]. Scoring adheres to the following criteria: (a) normal lung; (b) minimal fibrous thickening of alveolar or bronchiolar walls; (c) moderate thickening of walls without obvious damage to the lung architecture; (d) increased fibrosis with definite damage to the lung structure; (e) severe distortion of the structure and large fibrous areas; “honeycomb lung” is placed in this category; and (f) total fibrous obliteration of the field. The formation of fibrous bands or small fibrous masses corresponds to scores of 0, 1, 2, 3, 4, 5, and 6 and 7 and 8, respectively. The sections were viewed via an Olympus BX53 microscope at ×200 magnification.

Immunohistochemical and immunofluorescence staining were used to evaluate the severity of lung fibrosis. Microscopy images were assessed via ImageJ software to determine the integral optical density (IOD) and area of each image. The average optical density (AOD) was calculated via the formula AOD = IOD/area.

### 2.4 Hydroxyproline Assay and Hydroxylated Collagen Pyridine Assay Detected by ELISA

HYP assay: The collagen content in lung tissues was assessed with a hydroxyproline assay kit (Nanjing Jiancheng Bioengineering Institute). Approximately 50 mg of each tissue sample was combined with 1 mL of hydrolysate and immersed in a boiling water bath at 60°C for approximately 20 min. Following centrifugation of the supernatant at 3500 rpm for 10 min, the absorbance at 550 nm was measured. The HYP concentration was reported as μg/mg.

### 2.5 For the HLP Assay

50 mg of sample was weighed and placed in a test tube. Nine volumes of physiological saline were added at a ratio of weight (g):volume (mL) = 1:9. The tissue was cut into pieces, the homogenate was prepared in an ice bath, the homogenate was ultrasonicated at 5000 rpm, the mixture was centrifuged for 10 min, and the supernatant was collected for testing. The supernatants of A549 cells and MRC‐5 fibroblasts were collected at the grouped processing time points, the samples were centrifuged at 1000 rpm for 20 min, and the supernatants were collected for detection at 450 nm. The HLP concentration was reported as ng/mg.

### 2.6 Cell Culture and Treatment

Human primary lung fibroblasts (HPLF) were obtained from three patients who were diagnosed with IPF according to the 2022 IPF Clinical Practice Guideline [23]. All participants signed an informed consent form and were notified about the study prior to participation, and the study was approved by the West China Hospital of Sichuan University. The human type 2 alveolar epithelial cell line (A549), human embryonic lung fibroblasts (MRC‐5) and human primary lung fibroblasts (HPLF) were cultured in a sterile cell culture plate containing Dulbecco's modified Eagle's medium (DMEM) (Invitrogen, CA, USA) supplemented with 10% fetal bovine serum (FBS) (Gibco, Carlsbad, CA) and penicillin–streptomycin at 37°C in a 5% CO2 humidified incubator. The cells were dissociated via 0.25% trypsinization and plated in 12‐well plates at a density of 1 × 105 cells per well. The cells were treated with or without TGF‐β1 (10 ng/mL), SAB (50 μM) [15, 21], or vehicle (DMSO) for 48 h. To investigate the effect of LH2 on the β‐catenin‐dependent Wnt pathway in the epithelium and fibroblasts. When A549 cells, MRC‐5 and HPLF fibroblasts were passaged to 40%–50% confluence, medium containing short interfering RNA (siRNA) oligos against LH2 (si‐PLOD2, 5′‐3′CCACCAAGATTCTCCTGAATT) was added to the cells, or β‐catenin adenovirus (β‐catenin, 5′‐3′GCCACAAGATTACAAGAAA) was added to the cells. Six hours post‐transfection, 10% FBS DMEM containing 10 ng/mL TGF‐β1 alone was added for 48 h. These cells were subsequently harvested for further gene and protein detection.

### 2.7 Western Blot Analysis

Total protein was extracted from lung homogenates or cellular lysates via prechilled RIPA lysis buffer (Leagene Biotechnology, Beijing, China) containing a 1:100 dilution of phenylmethanesulfonyl fluoride (PMSF; Beyotime, Shanghai, China). Protein concentrations were quantified via a bicinchoninic acid (BCA) protein assay kit (Wanleibio Technology, Shenyang, China). The extracted protein samples were heated for approximately 10 min for denaturation. SDS–PAGE was employed to separate proteins (50 μg/lane), and PVDF membranes (Merck Millipore, Billerica, MA) were utilized for transfer. The membranes were incubated with primary antibodies overnight at 4°C. GAPDH was used as an internal control. After three 5 min washes with TBST, the membranes were subsequently incubated with horseradish peroxidase (HRP)‐conjugated secondary antibodies at room temperature for 1 h. Bands were visualized via an Fdbio‐pico electrochemiluminescence (ECL) kit (Fdbio Science, Hangzhou, China).

### 2.8 Public Data Analysis and Pharmacology Network Construction

To verify the expression of LH2, we obtained a public dataset, GSE169500, from the GEO database. These data were downloaded, and differentially expressed genes were reanalyzed via the DESeq2 package in R. Genes with adjusted *p* < 0.05 were considered differentially expressed, and heatmaps and volcano plots were generated with those DEGs.

For pharmacology network construction, we identified the targets of SAB via the herb database (http://herb.ac.cn/), with ‘Salvianolic acid B’ used as the search keyword. Simultaneously, IPF‐related targets were retrieved from the GeneCards database (https://www.genecards.org/) using ‘Idiopathic pulmonary fibrosis’ as the search keyword. The intersection targets between the IPF‐related genes and the predicted SAB targets were subsequently determined. These overlapping targets were ultimately imported into the GeneMANIA database (https://genemania.org/) to construct a comprehensive interaction network. Finally, through functional association network analysis of 23 candidate genes based on GeneMANIA. A total of 23 candidate genes were submitted to GeneMANIA to predict additional functionally related genes and their potential biological associations, and the network visualization was further optimized in Cytoscape. Inner‐circle nodes represent the 23 input genes, while outer‐circle nodes represent 23 additional genes related to the inputs. Node size indicates network connectivity, reflecting the extent of potential functional associations with other genes. Edges denote functional relationships between genes, suggesting their possible involvement in shared biological processes or regulatory networks, supported by evidence including co‐expression, physical interactions, predicted, genetic interactions, pathway, co‐localization, and shared protein domains.

### 2.9 Statistical Analysis

The experimental data were acquired from three independent trials and are presented as the means ± standard errors of the means (SEMs). All analyses were statistically assessed via SPSS22 software. One‐way ANOVA and Student's *t*‐test were used to evaluate the significance of differences between different groups. The analysis was performed via GraphPad Prism 10, and statistical significance was set at *p* < 0.05.

## 3 Results

### 3.1 LH2 Increased in Alveolar Epithelial Cells and Fibroblast During Pulmonary Fibrosis

**FIGURE 1:**
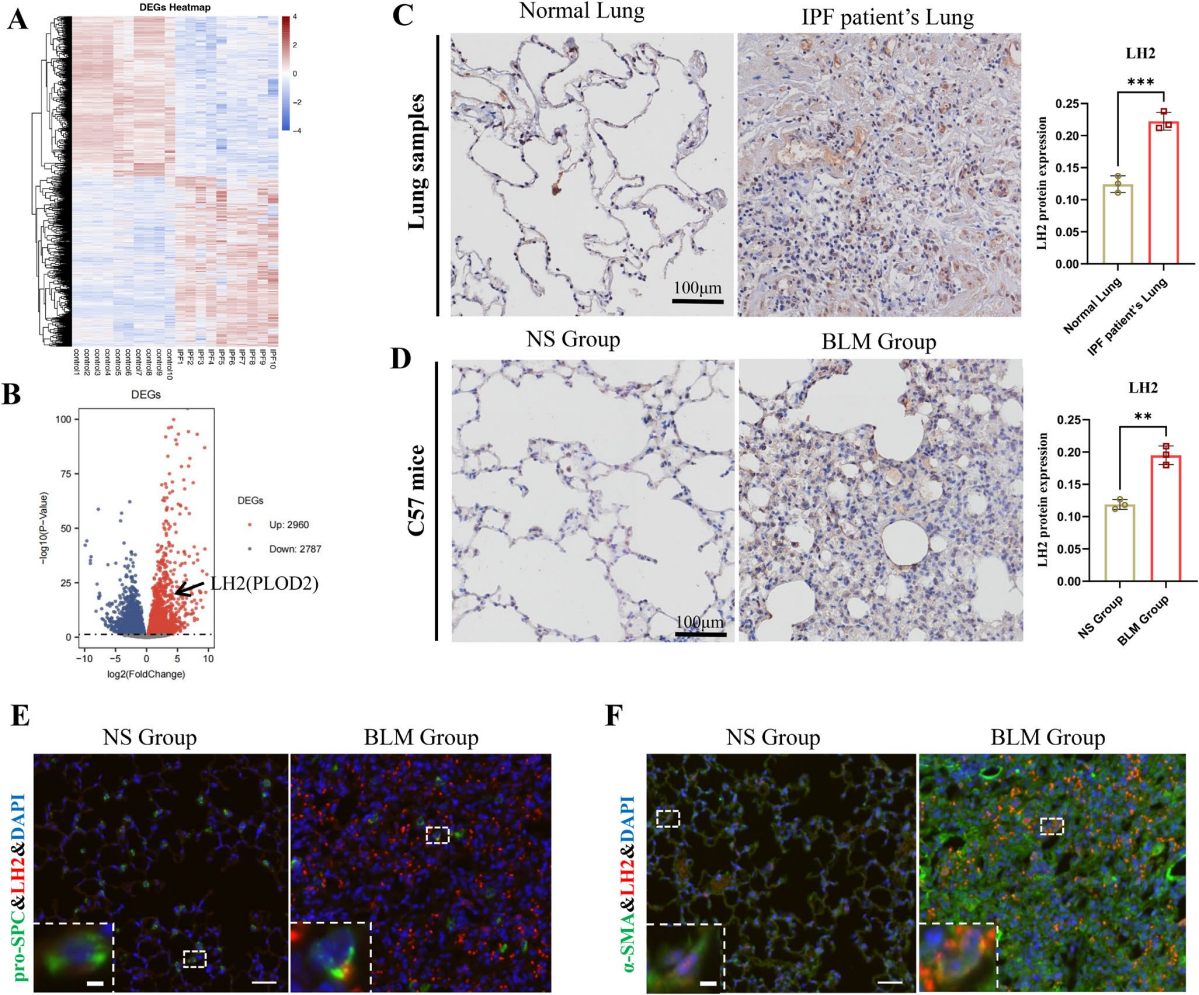
LH2 protein expression was significantly upregulated in pulmonary fibrosis tissues. (A, B) Bioinformatics analysis revealed that LH2 expression was elevated in IPF patients, highlighted by the black arrow. (C, D) Representative immunohistochemistry (IHC) images depicted LH2 expression, alongside quantified staining intensity, in lung biopsies from IPF patients and BLM‐exposed PF mice. Scale bar = 100 μm. (E, F) In lung biopsies from BLM‐exposed PF mice, immunofluorescence images depicted LH2 and pro‐SPC, LH2 and α‐SMA colocalization. Scale bar = 100 and 10 μm. Student's *t*‐test were used for statistical analysis, *n* = 3, *p < 0.05, **p < 0.01, ***p < 0.001.

To assess the role of lysyl hydroxylase 2 in pulmonary fibrosis, we first reanalyzed a publicly available RNA sequencing GEO dataset (GSE169500) comprising lung samples from IPF patients and controls. The mRNA of LH2 was markedly upregulated in lung tissues from patients with IPF compared with controls (Figure 1A,B). Immunohistochemical analysis of lung biopsies obtained from IPF patients showed significantly elevated LH2 protein levels relative to controls (Normal Lung) (*n* = 3 per group; Figure 1C). To further validate these findings, a murine model of pulmonary fibrosis was induced by intratracheal administration of bleomycin to wild‐type C57BL/6 mice. Consistent with the above observations, LH2 protein expression markedly increased in BLM‐induced fibrotic lungs compared with saline‐treated controls (NS Group) (Figure 1D). To investigate the localization of LH2 in fibrotic lungs, we further examined the LH2 protein level in alveolar epithelial cells and fibroblasts by co‐immunostaining with pro‐SPC and α‐SMA, respectively. As shown in Figure 1E,F, LH2 protein levels were significantly increased in both alveolar epithelial cells and fibroblasts from BLM‐induced PF mice compared with controls, indicating LH2 is involved in the pathogenesis of pulmonary fibrosis.

### 3.2 LH2 Silencing Attenuated TGF‐β1‐Induced Fibrotic Protein Expression

**FIGURE 2:**
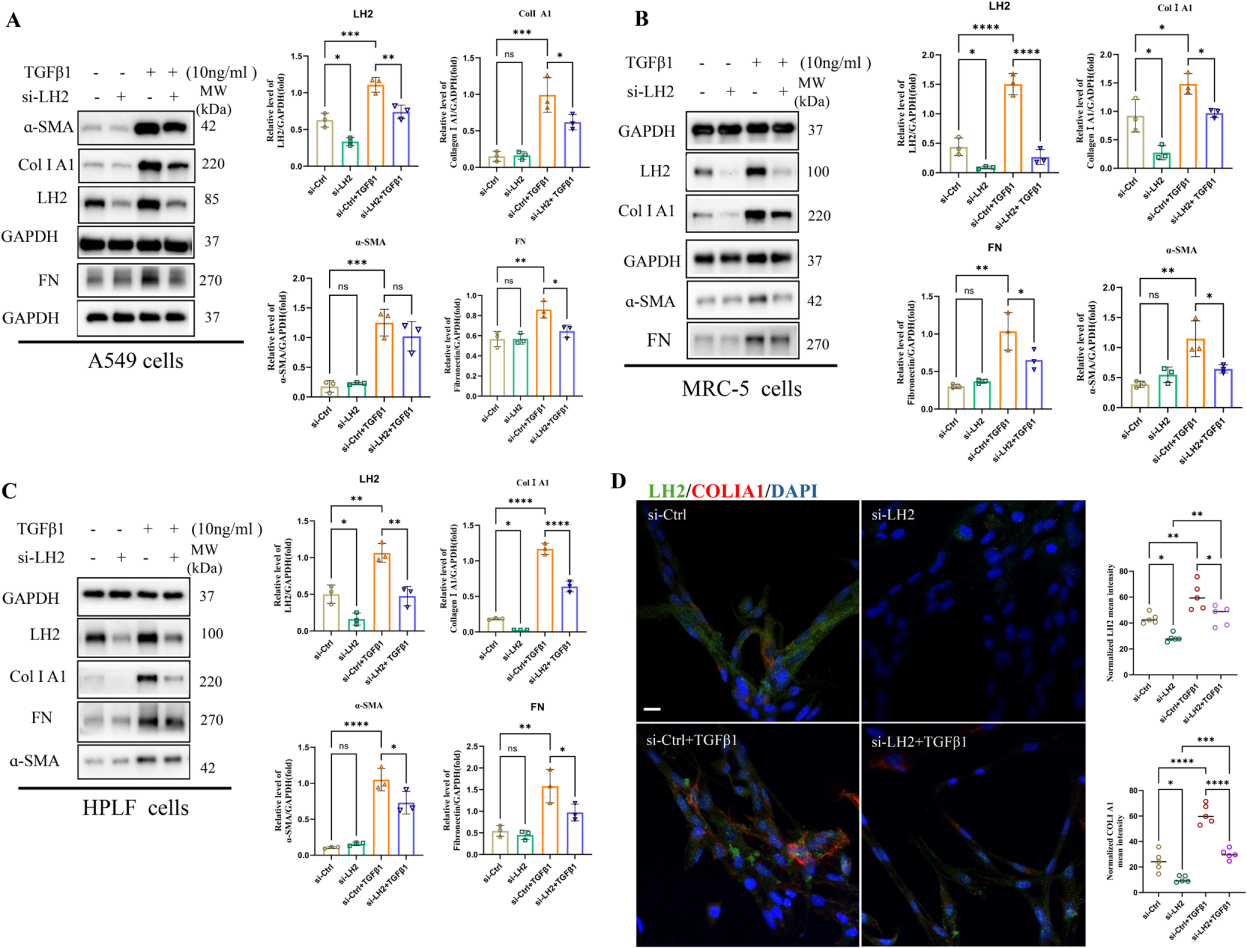
The antifibrotic effect of LH2 silencing in alveolar epithelial cells and pulmonary fibroblasts. (A) ECM protein (Col Ι A1, α‐SMA and FN) and LH2 expression in A549 cells were determined by immunoblotting and analysed via densitometry. One‐way ANOVA and Student's *t*‐test were used for statistical analysis, *n* = 3, *p < 0.05, **p < 0.01, ***p < 0.001, ns stands for nonsignificant difference. (B, C) ECM protein and LH2 expression in fibroblast cells (MRC‐5, HPLF) were determined by immunoblotting and analysed via densitometry. One‐way ANOVA and Student's *t*‐test were used for statistical analysis, *n* = 3, *p < 0.05, **p < 0.01, ***p < 0.001, ****p < 0.0001, ns stands for nonsignificant difference. (D) Representative confocal immunofluorescence images of LH2(green) and COLΙ A1(red) expression in MRC‐5 fibroblasts. Scale bar = 20 μm. One‐way ANOVA and Student's *t*‐test were used for statistical analysis, *n* = 3, *p < 0.05, **p < 0.01, ***p < 0.001, ****p < 0.0001.

Excessive deposition of extracellular matrix (ECM) proteins, particularly type I collagen, is a hallmark of pulmonary fibrosis (PF) [3]. To investigate the role of LH2 in pulmonary fibrosis, we performed siRNA‐mediated knockdown of LH2 in A549 epithelial cells, as well as MRC‐5 and HPLF fibroblasts, followed by stimulation with or without TGF‐β1 (10 ng/mL) for 48 h. As shown in Figure 2A–C, LH2 protein levels were efficiently reduced in the si‐LH2 group compared with the si‐Ctrl group, LOX expression slightly increased, whereas the expression of LH1 and LH3 remained unchanged (Figure S1). Under normal conditions, LH2 knockdown significantly decreased Col I A1 protein levels in MRC‐5 and HPLF fibroblasts, while FN and α‐SMA expression were not affected. In contrast, LH2 silencing did not alter the expression of Col I A1, FN, or α‐SMA in A549 epithelial cells. Upon TGF‐β1 stimulation, Col I A1, FN, and α‐SMA protein levels were markedly upregulated in both alveolar epithelial cells and fibroblasts. Notably, LH2 knockdown significantly attenuated TGF‐β1‐induced upregulation of these fibrotic ECM proteins in both cell types (Figure 2A–C). Similarly, the results of immunofluorescence staining revealed that the protein expression of LH2 and Col Ι A1 decreased to some extent after LH2 knockdown in MRC‐5 fibroblasts. However, LH2 knockdown significantly attenuated LH2 and Col Ι A1 expression upregulation in TGF‐β1‐induced MRC‐5 fibroblasts (Figure 2D). Thereby, the above results suggest that LH2 is required for the full activation of TGF‐β1‐induced fibrotic responses.

### 3.3 SAB Reduced LH2 Protein and Suppressed TGF‐β1‐Induced Fibrotic Protein Expression

**FIGURE 3:**
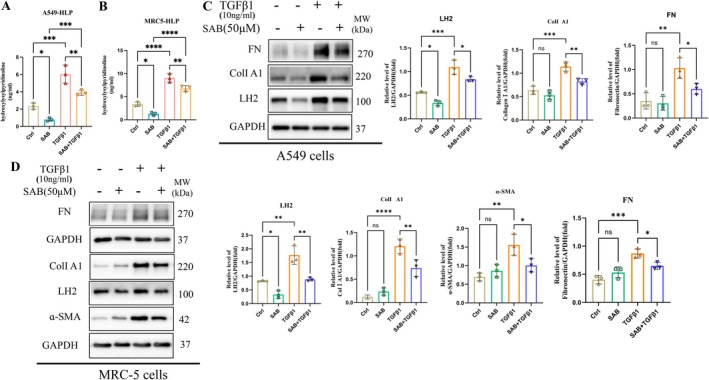
SAB suppressed HLP synthesis and ECM protein expression in TGF‐β1‐induced A549 cells and MRC‐5 cells. (A, B) HLP levels in the supernatants were measured in A549 and MRC‐5 cells. One‐way ANOVA and Student's *t*‐test were used for statistical analysis, *n* = 3, *p < 0.05, **p < 0.01, ***p < 0.001, ****p < 0.0001. (C) The protein levels of LH2, Col Ι A1 and FN in A549 cells were determined by immunoblotting and analysed via densitometry. One‐way ANOVA and Student's *t*‐test were used for statistical analysis, *n* = 3, *p < 0.05, **p < 0.01, ***p < 0.001, ****p < 0.0001, ns represents nonsignificant differences. (D) The protein levels of LH2, Col I A1, α‐SMA and FN in MRC‐5 fibroblasts were determined by immunoblotting and analysed via densitometry. One‐way ANOVA and Student's *t*‐test were used for statistical analysis, *n* = 3, *p < 0.05, **p < 0.01, ***p < 0.001, ****p < 0.0001, ns represents nonsignificant differences.

Our previous studies have revealed that LH2 plays a critical role in collagen post‐translational modification by catalysing the formation of hydroxylysylpyridinoline (HLP), thereby regulating collagen cross‐linking and extracellular matrix stability [14]. SAB, a major hydrophilic bioactive compound derived from *Salvia miltiorrhiza* , has been reported to exhibit anti‐inflammatory and anti‐fibrotic properties. To assess the effect of SAB on LH2 expression and fibrotic protein expression, ELISA and western blot analysis were performed in MRC‐5 and A549 cells. ELISA results showed that TGF‐β1 stimulation significantly increased HLP production, whereas SAB treatment markedly attenuated TGF‐β1‐induced HLP production (Figure 3A,B). Consistently, western blot analysis demonstrated that SAB reduced LH2 protein expression and suppressed TGF‐β1‐induced upregulation of fibrotic markers, including FN and α‐SMA in both A549 cells and MRC‐5 fibroblasts (Figure 3C,D). Together, these findings suggest that SAB exerts anti‐fibrotic effects that might be through inhibiting LH2 expression, thereby reducing HLP formation and attenuating ECM remodelling.

### 3.4 SAB Attenuated Bleomycin‐Induced Pulmonary Fibrosis in Mice

**FIGURE 4:**
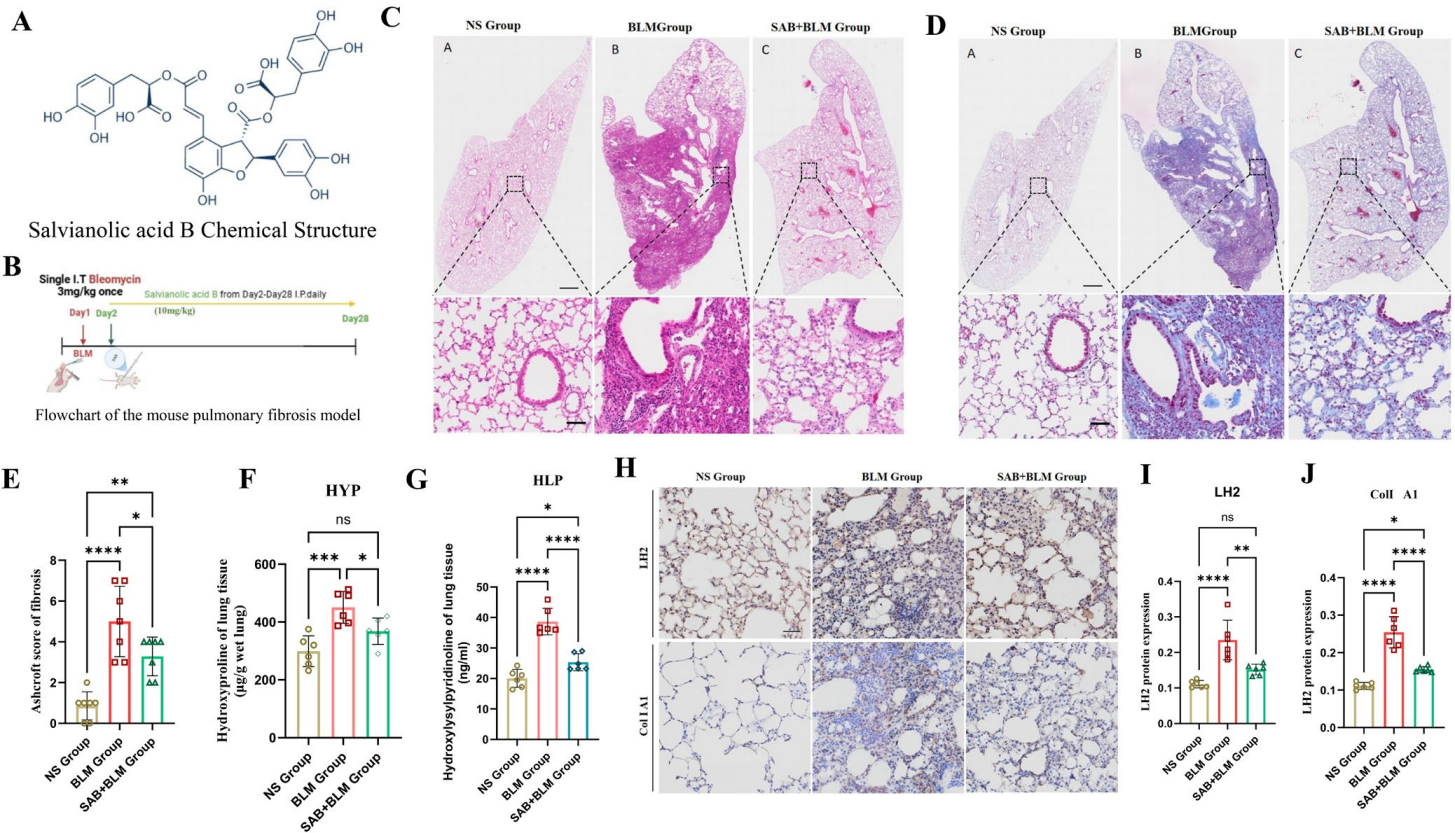
SAB alleviated BLM‐induced lung fibrosis in mice. (A) Chemical structure of SAB. (B) Flowchart of the pulmonary fibrosis mouse model. (C, D) Sections were stained with H&E and Masson's trichrome. Scale bar = 2 mm, and magnification: 20×. (E) The Ashcroft score of fibrosis was assessed via histological sections. (F–G) The total contents of HYP and HLP in mouse lung tissues were determined via a hydroxyproline kit and a hydroxylated collagen pyridine kit, respectively. (H–J) The protein levels of LH2 and ColI A1 were determined via IHC and analysed via densitometry. Scale bar = 50 μm. One‐way ANOVA and Student's *t*‐test were used for statistical analysis, *n* = 6, *p < 0.05, **p < 0.01, ****p < 0.0001, ns stands for nonsignificant difference.

Although SAB exhibits antifibrotic effects in vitro, whether it exerts similar effects in vivo remains unclear. To address this, we established a bleomycin (BLM)‐induced mouse model of pulmonary fibrosis via a single intratracheal instillation (Figure 4A,B). At 28 days post‐treatment, haematoxylin and eosin (H&E) staining revealed that BLM administration caused marked disruption of alveolar architecture, characterized by alveolar collapse, fusion, and structural remodelling (Figure 4C). Masson's trichrome staining further demonstrated that SAB treatment significantly reduced collagen deposition in lung tissues compared with the BLM group (Figure 4D). Consistently, SAB markedly alleviated fibrosis severity, as quantified by the Ashcroft score (Figure 4E).

BLM‐treated mice exhibited significantly elevated levels of hydroxyproline (HYP) and hydroxylysylpyridinoline (HLP) in lung tissue homogenates compared with the normal saline (NS) group, both of which were markedly reduced following SAB treatment (Figure 4F,G). Consistent with these observations, immunohistochemical (IHC) analysis revealed markedly increased LH2 and Col I A1 expression in the lungs of BLM‐treated mice, which was substantially attenuated upon SAB administration (Figure 4H–J). These findings demonstrate that SAB effectively reduces LH2 protein expression and attenuates bleomycin‐induced pulmonary fibrosis in vivo.

### 3.5 SAB Suppressed TGF‐β1‐Mediated EMT and FMT

**FIGURE 5:**
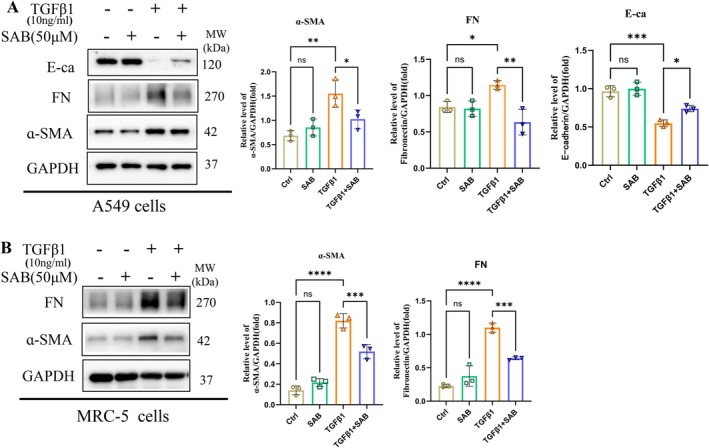
SAB inhibited EMT and FMT progression in TGF‐β1‐stimulated A549 cells and MRC‐5 fibroblasts, respectively. (A) The protein levels of E‐ca, α‐SMA, and FN in A549 cells were determined by immunoblotting and analysed by densitometry. One‐way ANOVA and Student's *t*‐test were used for statistical analysis, *n* = 3, *p < 0.05, **p < 0.01, ***p < 0.001, ns stands for nonsignificant difference. (B) The protein levels of α‐SMA and FN in MRC‐5 fibroblasts were determined by immunoblotting and analysed via densitometry. One‐way ANOVA and Student's *t*‐test were used for statistical analysis, *n* = 3, ***p < 0.001, ****p < 0.0001, ns stands for nonsignificant difference.

Epithelial‐to‐mesenchymal transition (EMT) and fibroblast‐to‐myofibroblast transition (FMT) play critical roles in pulmonary fibrosis, with TGF‐β serving as a key inducer of both processes [24, 25]. To evaluate the effect of SAB, A549 and MRC‐5 cell lines were stimulated with TGF‐β1 (10 ng/mL) to induce EMT and FMT, respectively. Downregulation of E‐cadherin (E‐ca) and upregulation of α‐SMA were used as core markers of EMT and FMT. Western blot analysis revealed that under basal conditions, SAB had no effect on α‐SMA, FN, or E‐cadherin levels in A549 cells. However, SAB reversed TGF‐β1‐induced increases in α‐SMA and FN, as well as the reduction in E‐cadherin (Figure 5A). In MRC‐5 fibroblasts, SAB decreased LH2 expression and suppressed TGF‐β1–induced upregulation of FN and α‐SMA. Together, these findings indicate that SAB simultaneously inhibits TGF‐β1–driven EMT and FMT in vitro (Figure 5B).

### 3.6 SAB Decreased β‐Catenin Protein Expression

**FIGURE 6:**
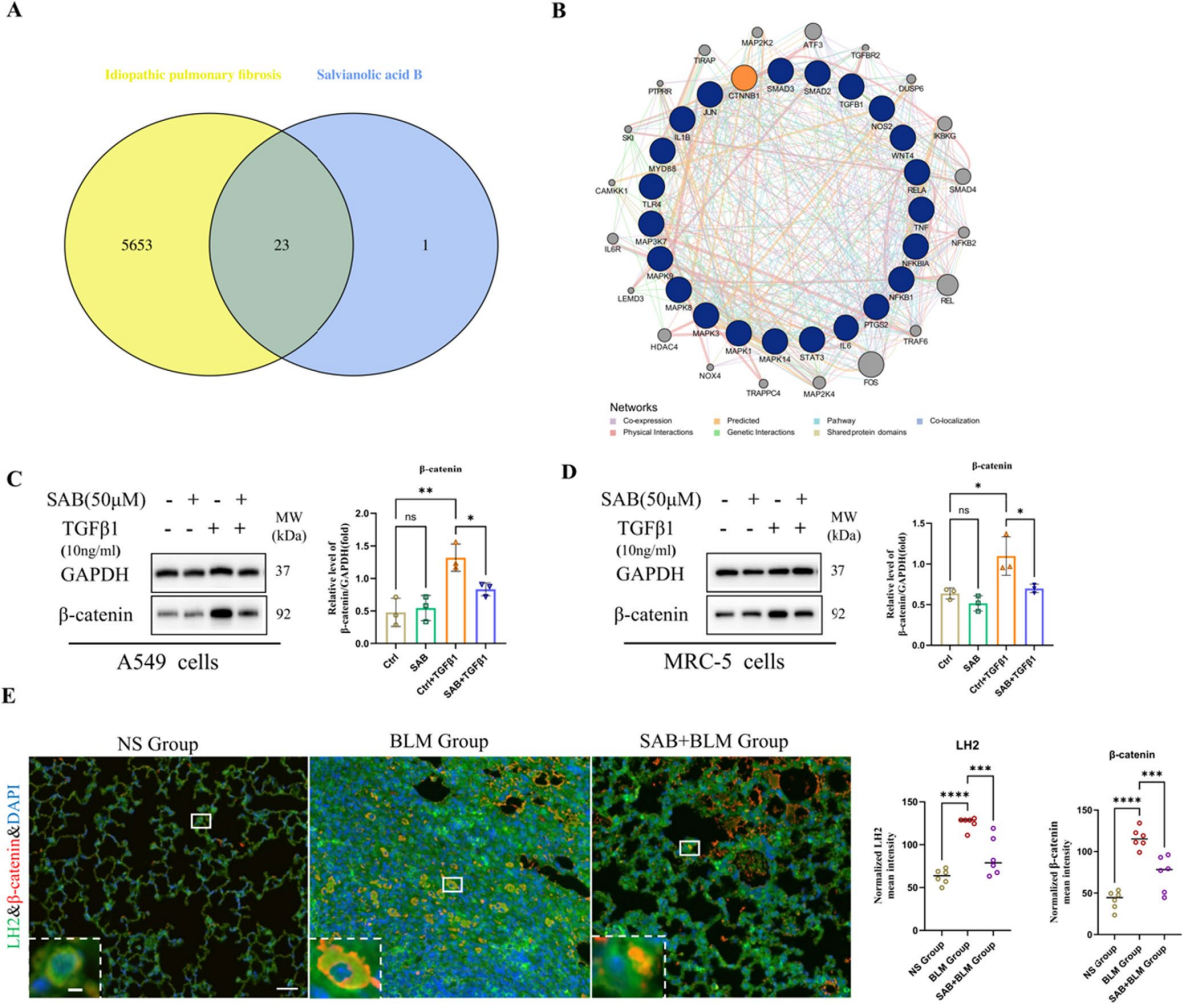
Network pharmacology analysis was used to predict SAB's potential signalling pathway targets for treating Pulmonary fibrosis. (A) Venn diagram depicting SAB targets and IPF‐related genes. (B) Cooperative gene network of 23 overlapping genes common to SAB targets and IPF‐related genes. (C, D) β‐catenin protein expression levels in A549 cells and MRC‐5 fibroblasts were determined by immunoblotting and analysed via densitometry. One‐way ANOVA and Student's *t*‐test were used for statistical analysis, *n* = 3, *p < 0.05, **p < 0.01, ns stands for nonsignificant difference. (E) Immunofluorescence analysis showed colocalization of β‐catenin(red) and LH2(green). Scale bars = 100 μm and 10 μm. Student's *t*‐test were used for statistical analysis, *n* = 6, ***p < 0.001, ****p < 0.0001.

The Wnt/β‐catenin signalling pathways are considered a key regulator of the pathogenesis of pulmonary fibrosis [19]. To investigate the specific regulatory pathways of genes overlapping between SAB targets and IPF‐related genes, we performed a network pharmacology analysis using Genemania. A total of 5676 IPF‐related genes were retrieved from the GenCards database, while 24 unique SAB target genes were obtained from the Herb database. The shared regulatory network revealed 23 overlapping genes between SAB and IPF (Figure 6A). Subsequently, the GeneMANIA analysis of the 23 overlapping genes indicated that SAB is associated with Wnt/β‐catenin, TGF‐β/Smad, and MAPK signalling pathways (Figure 6B). To validate the findings, we using western blot to detect the protein level of β‐catenin (encoded by CTNNB1). The results demonstrated that SAB inhibits TGF‐β induced β‐catenin protein expression (Figure 6C,D). Immunofluorescence analysis revealed pronounced colocalization of β‐catenin (red) and LH2 (green) in the fibrotic regions of lung tissues from BLM‐exposed mice. Quantitative analysis showed that, compared with the NS group, fluorescence intensities of both β‐catenin and LH2 were significantly increased in the BLM group, and these elevations were markedly attenuated upon SAB treatment (Figure 6E). These data suggest a potential relationship between SAB, the Wnt/β‐catenin signalling pathway, and LH2.

### 3.7 β‐Catenin Silencing Attenuated TGF‐β1‐Induced Fibrotic Protein Expression

**FIGURE 7:**
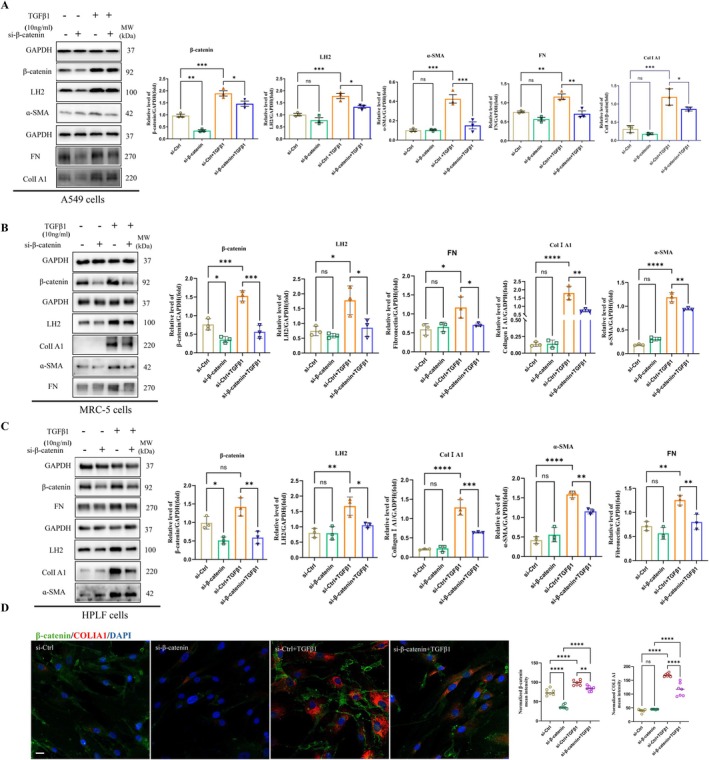
The antifibrotic effect of si‐β‐catenin in A549 cells and lung fibroblasts. (A–C) β‐catenin and ECM(Col Ι A1, α‐SMA and FN) protein expression levels in A549 cells and fibroblasts (MRC‐5, HPLF) were determined by immunoblotting and analysed via densitometry. One‐way ANOVA and Student's *t*‐test were used for statistical analysis, *n* = 3, *p < 0.05, **p < 0.01, ***p < 0.001,****p < 0.0001, ns stands for nonsignificant difference. (D) Representative confocal immunofluorescence images of β‐catenin (green), Col Ι A1 (red) and DAPI (blue) in MRC‐5 cells. Scale bars = 20 μm. One‐way ANOVA and Student's *t*‐test were used for statistical analysis, *n* = 3, **p < 0.01, ****p < 0.0001.

To elucidate the role of Wnt/β‐catenin signalling in regulating LH2 and fibrotic protein expression, we performed siRNA‐mediated knockdown of β‐catenin in A549 epithelial cells, as well as MRC‐5 and HPLF fibroblasts, followed by stimulation with or without TGF‐β1 (10 ng/mL) for 48 h. As shown in Figure 7A–D and Figure S2, under basal conditions, silencing of β‐catenin increased GSK‐3β expression but had no significant effect on the protein levels of LH2, FN, α‐SMA, or COL1A1. In contrast, β‐catenin knockdown markedly attenuated TGF‐β1–induced upregulation of LH2, FN, α‐SMA, COL1A1. These findings suggest that Wnt/β‐catenin signalling may cooperate with LH2 to regulate fibrotic responses under pathological conditions.

## 4 Discussion

Structural alterations characterized by tissue remodelling and fibrosis represent significant hallmarks of numerous pulmonary disorders, including asthma, chronic obstructive pulmonary disease and IPF [26]. Research suggests that the hallmark of fibrosis is abnormal collagen accumulation in the ECM, ultimately leading to organ failure [8, 27]. Therefore, changes in the collagen accumulation of ECM are potential targets for the treatment of fibrotic diseases. To date, there has been a lack of focus on the development of pharmacological agents aimed at the ECM, despite its critical involvement in the advancement of these diseases.

Far from being a passive filler, the ECM functions as a dynamic and spatially organized ‘command centre’ that orchestrates cell behaviour. Structural and compositional remodelling of the ECM serves as a key determinant in directing cells toward tissue repair or fibrotic responses [28]. Excessive accumulation of collagen in the ECM is the final stage of disease‐related tissue injury and repair [5, 8, 28]. During biosynthesis, collagens undergo extensive PTMs to fold into right‐handed triple helices. Among these modifications, proline hydroxylation is the most studied and understood. It is essential for the formation and stabilization of the collagen right‐handed triple helix [29, 30]. In the BLM‐induced PF mouse model, we also detected an increase in hydroxyproline (Figure 4E). PTMs increase hydroxylated collagen cross‐linking, leading to stabilization of the collagen network and limiting fibrosis reversibility [1, 9, 11]. Not only did the level of cross‐linking increase, but the composition also changed; specifically, an increase in hydroxylysine‐derived cross‐links in fibrotic tissues was observed [11]. In this study, we explored the effects of HLP on the synthesis of extracellular matrix collagen in pulmonary fibrosis. Interestingly, HLP levels were significantly upregulated in TGF‐β1–treated A549 cells (Figure 3A) and MRC5 fibroblasts (Figure 3B), and in bleomycin‐induced mouse models (Figure 4F). In parallel, the increase of HLP is closely related to PTMs.

At present, two enzyme families are responsible for the cross‐linking modification of collagen: lysyl oxidase (LOX) family members (LOX, LOXL1, LOXL2, LOXL3, and LOXL4) [31] and lysyl hydroxylase family members (LH1, LH2, and LH3) [32]. These enzymes represent crucial groups of enzymes involved in the PTMs of collagen proteins. Previous studies have reported that inhibition of the LOX family as a whole, specifically of LOX, LOXL1, and LOXL2, suppressed fibrosis progression and accelerated its reversal in rodent models of cardiac, renal, pulmonary, and liver fibrosis. However, recent disappointing clinical trials with a monoclonal antibody against LOXL2 (simtuzumab) in patients with pulmonary and liver fibrosis have dampened enthusiasm for LOX family member inhibition [31, 33]. This unexpected negative outcome may be related to other PTMs enzymes. Wanhui Dong reported that hydroxylation is essential for collagen maturation [34]. This results in irreversible fibrosis, as hydroxylated collagen cross‐linking is more difficult to degrade. LH2, the enzyme responsible for the process of hydroxylation, is universally upregulated in fibrosis [11]. Inhibition of this enzyme is expected to lead to reversible fibrosis without interfering with the normal repair process. Thus, we explored the influence of LH2 on collagen cross‐linking. We observed high LH2 expression in the lung tissues of IPF patients and BLM‐induced PF mice model (Figure 1A,B). To further understand the mechanism of LH2 upregulation in pulmonary fibrosis, we first selectively silenced LH2 gene in MRC‐5 cells, and found that it had no effect on the other subtypes, LH1, LH3, but LOX expression was increased (Figure S1). Next, we further silenced LH2 in A549 cells and fibroblasts (MRC‐5,HPLF), respectively. The results revealed that the protein expression of Col I A1 decreased in fibroblasts (MRC‐5 and HPLF), whereas there was no change in A549 cells. The fibrotic proteins (α‐SMA and FN) expression remained unchanged in both alveolar epithelial cells and fibroblasts. However, the expression of LH2 and fibrotic proteins significantly increased after TGF‐β1 treatment (Figure 2). These results confirmed that LH2 promoted fibrosis by TGF‐β1‐induced activation of fibroblasts and increasing collagen synthesis in the ECM.

Currently, the only pharmacotherapies with a grade‐A evidence base in IPF are pirfenidone, nintedanib, and nerandomilast. These compounds slow—but do not halt or reverse—parenchymal remodelling, reducing the annual rate of FVC decline by approximately 50%. Their clinical utility is constrained by dose‐limiting gastrointestinal, hepatic, and cutaneous adverse events that lead to dose reduction or discontinuation in 20%–30% of patients [4, 35, 36]. Active components derived from traditional Chinese medicines (TCM) modulate fibrogenesis through complementary, multi‐target mechanisms, including SAB [13, 37]. Previous studies have demonstrated that SAB could alleviate the fibrosis progression of multiple organs including the liver, kidney, and lung, and its main mechanism of antifibrosis is concentrated in the aspects of antioxidative stress and antiinflammation [18, 21, 38, 39, 40]. However, the pharmacological effects and mechanisms of SAB in PF treatment also remain unclear. In this study, we explored the mechanism by which SAB prevented pulmonary fibrosis by influencing extracellular matrix collagen cross‐linking synthesis, which had not been reported yet. After a series of experiments, we demonstrated that SAB attenuated BLM‐induced PF by decreasing HLP synthesis in the ECM (Figures 3A and 4D,G). Collectively, these results demonstrated that SAB mitigated pulmonary fibrosis in BLM‐induced mice through the suppression of collagen deposition and fibrosis‐related protein expression in the lung (Figure 4).

Fibroblast activation, characterized by their transformation into myofibroblasts, is a central event in tissue fibrosis. This process drives the excessive deposition of ECM and the secretion of various profibrotic mediators, leading to scar formation and organ dysfunction [1, 3]. In terms of phenotypic changes, the injured epithelial cells produce profibrotic cytokines that induce EMT and the activation of pulmonary fibroblasts, which are the major sources of myofibroblasts. To clarify the therapeutic target of SAB, we selected A549 cells and MRC‐5 fibroblasts to evaluate the in vitro effects of SAB. Rock's team followed ATII cell fate in a bleomycin‐induced murine transgenic model of pulmonary fibrosis and reported no transition of labelled ATII cells into myofibroblasts [41]. The occurrence of EMT in the PF model is controversial, as lineage tracing studies have reported conflicting results, either supporting or denying a pathogenetic role for EMT in lung fibrosis [42]. Even so, most scholars still believe that EMT is a critical stage during PF development and plays an important role at present. TGF‐β1 is a key regulator of EMT, as we described previously. E‐cadherin is a biomarker of epithelial cells, while α‐SMA is considered a hallmark of fibroblasts [43]. In vitro, we observed that SAB treatment significantly suppressed EMT in alveolar epithelial cells, as demonstrated by alterations in the expression of differentiation markers (such as E‐cadherin and α‐SMA). As shown in Figure 5A, E‐ca protein expression was downregulated and α‐SMA was upregulated after TGF‐β1 treatment, indicating that TGF‐β1 induced EMT in A549 cells. SAB treatment increased E‐ca protein expression and decreased α‐SMA protein expression. Liu reported that Salvianolic acid B inhibits myofibroblast transdifferentiation in experimental pulmonary fibrosis via the upregulation of Nrf2 [44]. As shown in Figure 5B, α‐SMA and FN protein expression increased upon TGF‐β1 treatment in MRC‐5 fibroblasts, suggesting that fibroblasts differentiated into myofibroblasts, but α‐SMA and FN protein expression decreased after SAB treatment. Thereby, these current data reveal that SAB suppresses the process of TGF‐β1‐driven EMT and FMT.

Among the many profibrotic factors that have been identified, TGF‐β and Wnts that drives fibroblast activation and organ fibrosis in a variety of disease settings [19, 45]. Common lung diseases are closely associated with the Wnt/β‐catenin signalling pathway [24]. Activation of this pathway occurs in the lung epithelium of patients with IPF [1, 27]. In IPF, Wnt signalling is abnormally activated, characterized by significantly elevated expression levels of Wnt1, Wnt3a, Wnt10b, FZD2/3, β‐catenin, and LEF1 within lung tissue, consequently promoting fibroblast proliferation and EMT [46]. This abnormal activation stimulates mesenchymal cell proliferation and facilitates the fibrotic process [1, 7, 47]. Voloshanenko reported Wnt/β‐catenin is required for regulation of non‐canonical Evi/Wls target genes. In colon cancer cells Silenced Evi/Wls, mRNA expression of PLOD2(LH2) was downregulated [48]. Review on transcriptional regulation of Wnt/β‐catenin in colorectal cancer, explicitly summarizes PLOD2 as a non‐canonical Wnt target regulated by WNT5A/B [49]. Notably, PLOD2 has been reported as a non‐canonical Wnt‐responsive target in colorectal cancer rather than a universally established direct canonical β‐catenin target. Consistent with previous reports, β‐catenin has been identified as a key mediator of fibrogenesis, and its aberrant activation is a defining feature of IPF. An analysis of the gene expression profiles of healthy and IPF lung tissues (GSE169500) and a network pharmacology investigation of salvianolic acid B was regulated by the Wnt signalling pathway (Figure 6A,B). In addition, L1CAM, a known β‐catenin/TCF target, has been shown to induce PLOD2 expression, supporting the possibility that PLOD2 may act as a secondary downstream effector within β‐catenin‐activated transcriptional programmes [50]. On the other hand, direct transcriptional regulation of PLOD2 has also been linked to TGF‐β1/SMAD3/SP1 [51]. Meanwhile, to further address this point without overinterpretation, we additionally surveyed public resources for transcriptional and regulatory evidence linking Wnt signalling to PLOD2/LH2, including TF binding databases (e.g., ChIP‐Atlas/Cistrome DB), motif prediction resources (JASPAR), and public transcriptomic datasets involving Wnt pathway perturbation. Collectively, these resources support a regulatory association between Wnt signalling and PLOD2, but do not yet provide sufficient evidence to define PLOD2 as a universal direct canonical β‐catenin target. Immunofluorescence results demonstrated pronounced colocalization of β‐catenin and LH2, SAB intervention markedly attenuated the expression of both β‐catenin and LH2(Figure 6E). To further elucidate its mechanism in lung fibrosis, β‐catenin was silenced in alveolar epithelial cells and fibroblasts via β‐catenin‐specific adenovirus (si‐β‐catenin), Silencing β‐catenin in A549 cells and fibrobasts (MRC‐5, HPLF) slight changed LH2 expression, whereas there was no statistical significance (Figure 7A). Notably, upon TGF‐β1 stimulation for 48 h, LH2 and fibrotic protein expression increased; but in the si‐β‐catenin + TGF‐β1 group, fibronectin expression is dramatically decreased. These results suggested that TGF‐β1 may induce Wnt signalling pathway activation. While upstream Wnt ligand expression remained unchanged, dissociation of the GSK3‐β‐containing destruction complex promoted β‐catenin stabilization and accumulation, indicated activation of Wnt/β‐catenin signalling pathway (Figure S2). Lv also reported a close interconnection between these pathways [19]. Although publicly available evidence and our data supports that LH2/PLOD2 is functionally connected to Wnt/β‐catenin signalling pathway, but current evidence does not robustly establish PLOD2 as a canonical direct β‐catenin target across contexts. Instead, LH2 appears to be regulated in a context‐dependent manner, potentially as an indirect downstream effector of β‐catenin‐driven programmes and/or through non‐canonical Wnt signalling. Hence, silencd β‐catenin likely inhibits LH2 protein expression by suppressing Wnt pathway activation, thereby reducing fibrotic protein expression of ECM and ameliorating pulmonary fibrosis.

In addition to its mechanistic role, SAB exhibits notable advantages in clinical translation. As a bioactive compound derived from *S. miltiorrhiza* , SAB has been widely used in cardiovascular and inflammatory diseases with a well‐established safety profile and favourable pharmacokinetic properties [52, 53, 54]. Furthermore, lysyl hydroxylase 2, a key enzyme regulating collagen cross‐linking and ECM stabilization, has emerged as a critical driver of fibrotic progression. Increased LH2 expression is strongly associated with excessive collagen deposition and tissue stiffening in pulmonary fibrosis. Targeting LH2 may therefore represent a novel therapeutic strategy by disrupting pathological collagen maturation rather than merely inhibiting collagen synthesis. In this context, our findings that SAB suppresses LH2 expression suggest a dual mechanism involving both attenuation of collagen production and modulation of ECM remodelling. Collectively, these results highlight the potential of LH2 as a therapeutic target and support the translational value of SAB in the treatment of pulmonary fibrosis. Recent studies have demonstrated its anti‐fibrotic effects across multiple organ systems, largely attributed to its antioxidant, anti‐inflammatory, and ECM‐modulating activities. These characteristics make SAB a promising candidate for repurposing in pulmonary fibrosis. SAB dry powder inhaler for the treatment of idiopathic pulmonary fibrosis [55]. Phospholipids of inhaled liposomes determine the in vivo fate and therapeutic effects of SAB on idiopathic pulmonary fibrosis [56]. Such findings will be beneficial to the development of inhalable lipid‐based nanodrug delivery systems for the treatment of respiratory diseases where inhalation is the preferred route of administration.

## 5 Conclusion

**FIGURE 8:**
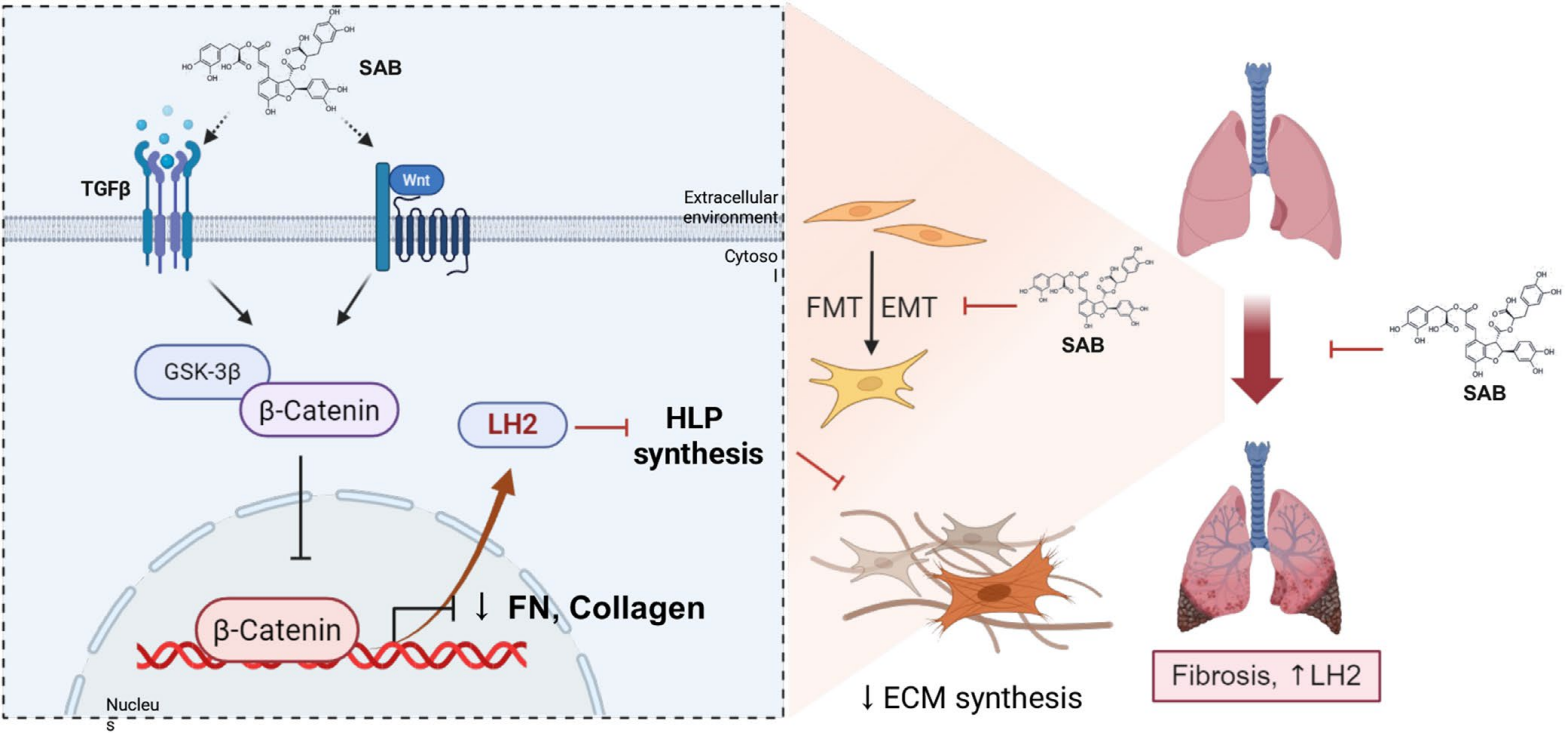
The potential mechanism of SAB in anti‐fibrosis.

In conclusion, SAB alleviated pulmonary fibrosis, at least in part, through inhibition LH2 expression and hydroxylated collagen cross‐linking synthesis processes, as well as key profibrotic processes, including EMT, FMT, and Wnt/β‐catenin signalling pathway (Figure 8).

## 6 Limitations of Study

Although our findings indicate that the antifibrotic effect of SAB may be attributed to its inhibition of the Wnt/β‐catenin signalling pathway, as well as to the attenuation of LH2 protein expression, we can't exclude other mechanisms or effects of SAB. Additionally, we don't create genetically modified mice with selective knockout of LH2 but directly observe the effect of LH2 on BLM‐induced pulmonary fibrosis in vivo. In the future, we will continue to observe the intervention effects and mechanisms of SAB on pulmonary fibrosis in a more detailed and dynamic manner. Moreover, further investigation is needed to clarify the mechanistic link between Wnt/β‐catenin signalling and LH2 regulation. Future studies employing chromatin immunoprecipitation (ChIP) and luciferase reporter assays will help determine whether β‐catenin directly regulates LH2 transcription at the promoter level.

## Author Contributions

**Songjun Shao:** investigation, conceptualization, data curation, methodology, writing – original draft. **Shanshan Rao:** data curation, investigation, methodology, validation. **Silu Hu:** investigation, formal analysis, methodology, software. **Yabin Zhang:** investigation, data curation, methodology. **Peng Zheng:** investigation, methodology. **Chunjie Li:** investigation, methodology. **Lichun Zhong:** methodology. **Yunzhi Long:** methodology. **Xin Tian:** methodology. **Xiaoju Tang:** writing – review and editing, project administration, methodology. **Fengming Luo:** resources, conceptualization, funding acquisition, visualization.

## Funding

The present study was funded by grants from the National Natural Science Foundation of China (82470062), the Science and Technology Program of Guizhou Province (ZK[2021]‐346), the Health Commission of Guizhou Province (gzwjkj grant No. 2021‐1‐100), and the Postdoctor Research Fund of West China Hospital, Sichuan University (2024HXBH116).

## Conflicts of Interest

The authors declare no conflicts of interest.

## Supporting information


**Figure S1:** LH2 knockdown modulated LH family members and LOX expression in MRC‐5 fibroblasts. siRNA‐mediated LH2 knockdown in MRC‐5 fibroblasts selectively increased LOX expression while leaving LH1 and LH3 unchanged. One‐way ANOVA and Student's *t*‐test were used for statistical analysis, *n* = 3, *p < 0.05, **p < 0.01, ns stands for nonsignificant difference.

**Figure S2:** β‐catenin knockdown modulated Wnt signalling members expression in pulmonary fibroblasts. (A, B) Adenovirus‐mediated β‐catenin knockdown in pulmonary fibroblasts (MRC‐5 and HPLF) selectively increased GSK3‐β expression while leaving Wnt3a unchanged. One‐way ANOVA and Student's *t*‐test were used for statistical analysis, *n* = 3, *p < 0.05, ns stands for nonsignificant difference.

## Data Availability

The data that support the findings of this study are available from the corresponding author upon reasonable request.
