# Supplementary figures and images for: Salvianolic Acid B Inhibited LH2 Expression to Reduce Collagen Synthesis in Pulmonary Fibrosis
Source: J Cell Mol Med. 2026 May 25;30(10):e71168. doi: 10.1111/jcmm.71168 (PMC13240261; doi:10.1111/jcmm.71168)

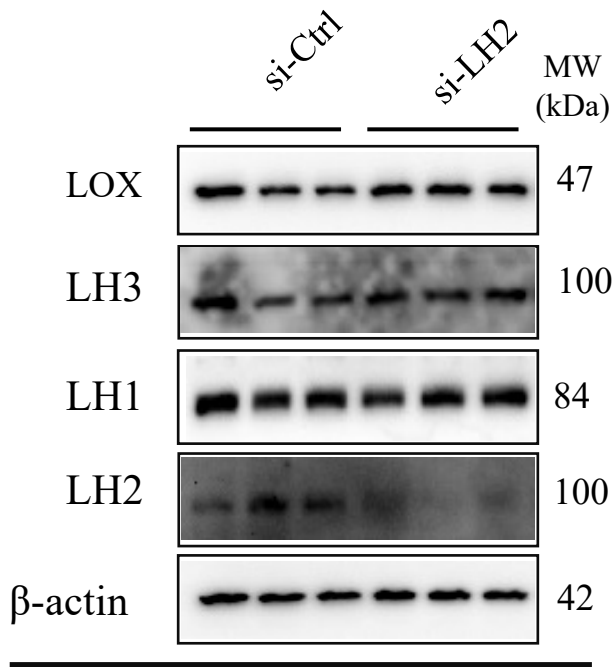

MRC-5 cells

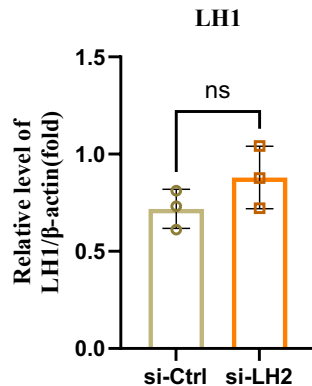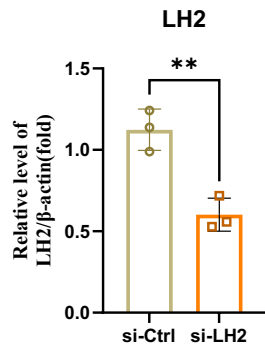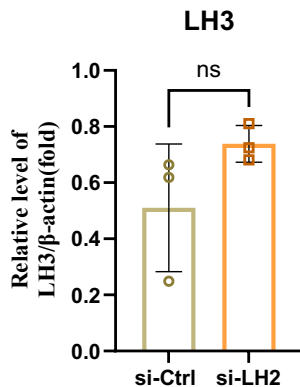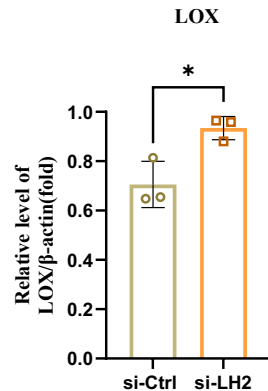

Supplement: Supplementary file 1 — Figure S1: LH2 knockdown modulated LH family members and LOX expression in MRC‐5 fibroblasts. siRNA‐mediated LH2 knockdown in MRC‐5 fibroblasts selectively increased LOX expression while leaving LH1 and LH3 unchanged. One‐way ANOVA and Student's t‐test were used for statistical analysis, n = 3, *p < 0.05, **p < 0.01, ns stands for nonsignificant difference. [file JCMM-30-e71168-s001.pdf]

**A**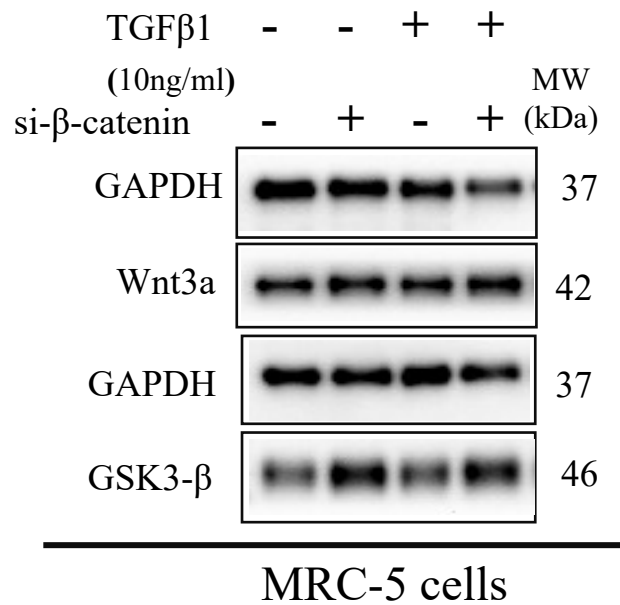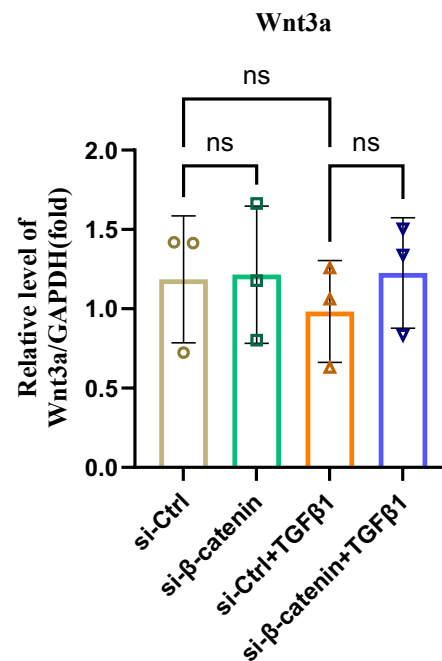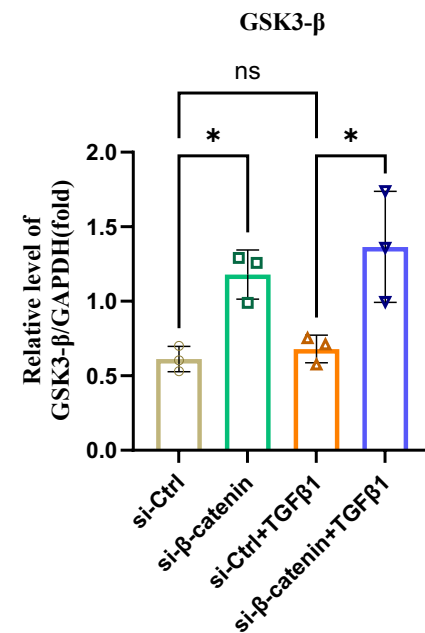**B**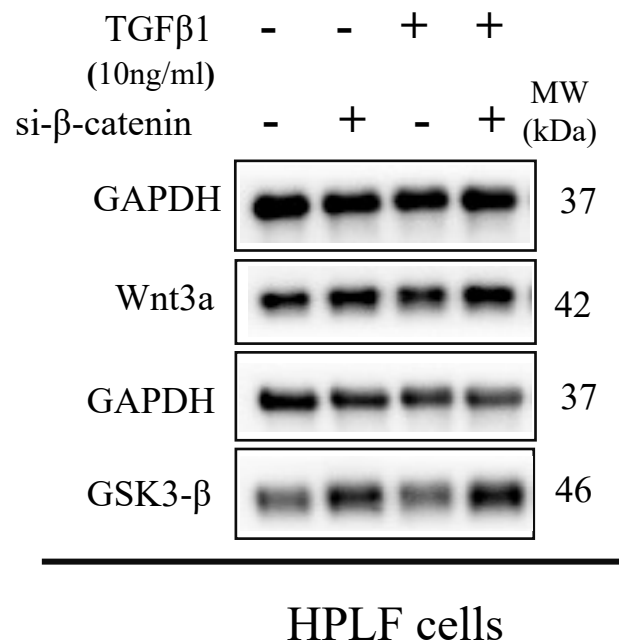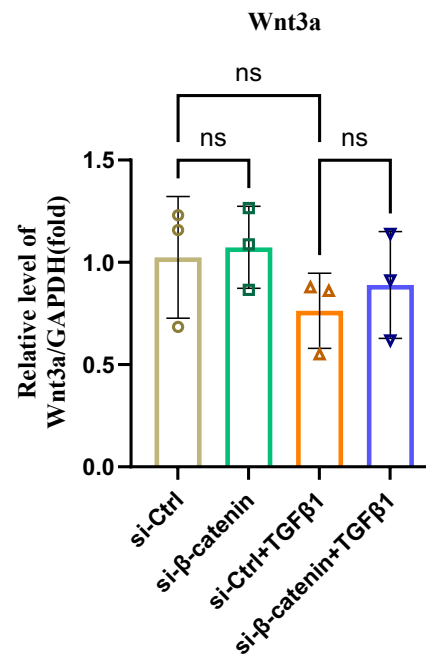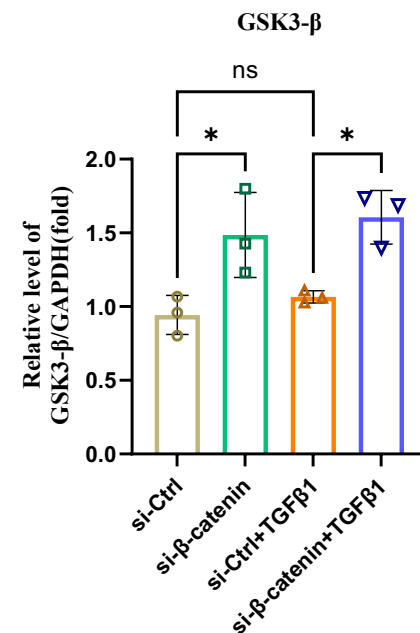

Supplement: Supplementary file 2 — Figure S2: β‐catenin knockdown modulated Wnt signalling members expression in pulmonary fibroblasts. (A, B) Adenovirus‐mediated β‐catenin knockdown in pulmonary fibroblasts (MRC‐5 and HPLF) selectively increased GSK3‐β expression while leaving Wnt3a unchanged. One‐way ANOVA and Student's t‐test were used for statistical analysis, n = 3, *p < 0.05, ns stands for nonsignificant difference. [file JCMM-30-e71168-s002.pdf]
